# Oxidative Stress in Veterinary Medicine

**DOI:** 10.4061/2011/812086

**Published:** 2011-08-17

**Authors:** Cristina Castillo Rodríguez, Fernando Wittwer Menge, José Joaquin Cerón

**Affiliations:** ^1^Departamento de Patología Animal, Facultad de Veterinaria de Lugo, Universidad de Santiago de Compostela, 27002 Lugo, Spain; ^2^Instituto de Ciencias Clínicas Veterinarias, Universidad Austral de Chile, Casilla 567, Valdivia, Chile; ^3^Departamento de Medicina y Cirugía Animal, Facultad de Veterinaria, Universidad de Murcia, Campus de Espinardo, Espinardo, 30100 Murcia, Spain

Oxidative stress can be regarded as an imbalance between prooxidant/free radical production and opposing antioxidant defenses. There is growing evidence that oxidative stress (OS) significantly impairs organic function and plays a major role in the aetiology and pathogenesis of several metabolic diseases in veterinary medicine. In many of these cases, it is unclear if oxidants trigger the disease or if they are produced as a secondary consequence of the disease and from general tissue damage.

In this special issue on oxidative stress in veterinary medicine, we have invited a few papers that address novel approaches about this matter, taking into account not only the pathogenic mechanisms of diseases but also new specific laboratorial tools helping in the OS measurement.

The first paper on this special issue addresses the advantages of measuring hepatic oxidative status in liver biopsy, helping in diagnosis of hepatic dysfunction and reflecting the degree of deterioration in the liver tissues. Thus, liver biopsy aids in recommending antioxidant's therapy in patients that had a hepatic disease with derangement in hepatic antioxidant constituents.

On the other hand, an increasing body of evidence suggests that OS is involved in the pathogenesis of a wide range of cardiovascular diseases. Nevertheless, it is still a matter of debate whether this increased OS has a primary causative role in cardiovascular disease pathogenesis or rather is a vascular sequel of disease progression. The establishment of the specific role of OS in cardiovascular diseases will help to choose the antioxidant therapy that will prove beneficial in combating these problems. The second paper performs a wide revision regarding the pathogenesis of OS and cardiac diseases in dog, and how supplementation can play a protective role, avoiding cell disorganisation and cellular damages. The authors describe there the effect of proper antioxidant supplementation (coenzyme-Q10, polyphenols, or omega-3 fatty acids) increasing the concentration of antioxidants in heart cells and making them less sensitive to free radicals.

Finally, a number of vitamins and trace minerals are involved in the antioxidant defense system and a deficiency of any of these nutrients may depress immunity. Some vitamins (such as E or C) are important antioxidants that have been shown to play an important role in immunoresponsiveness and health. A number of trace minerals are required for functioning of enzymes involved in the antioxidant defense system, and certain trace minerals may also affect immune cells via mechanisms distinct from antioxidant properties. Two reports analyze the protective effects of Zn or vitamin C in different species (chickens and mice) in different diseases (parasitic infections and haematological disturbances).

Finally, OS has been implicated in the pathogenic mechanism of some heavy metals (such as lead or cadmium), causing many disease conditions and toxicities in animals. Several ameliorative measures to counteract the oxidative damage to the body system aftermath or during exposure to these toxicants have been assessed with the use of antioxidants. The last report focuses on this aspect. 

This is a novel field of research and it is expected an increased number of studies in the future. 

*Cristina Castillo Rodríguez* *Cristina Castillo Rodríguez* *Fernando Wittwer Menge* *Fernando Wittwer Menge* *José Joaquin Cerón* *José Joaquin Cerón*

